# Morphologic and genomic changes of thyroid cancer cell lines exposed to conditions of simulated microgravity

**DOI:** 10.1038/s41526-024-00346-y

**Published:** 2024-01-15

**Authors:** Jong-hyuk Ahn, Sungyeon Park, Young Mi Hwang, Yun Suk Choi, Jin Wook Yi

**Affiliations:** 1 Department of Surgery, lnha University College of Medicine, Incheon, Korea; 2 Department of Surgery, Chungbuk National University Hospital, Cheongju, Korea; 3 College of Medicine, The Inha University of Korea, Incheon, Republic of Korea; 4 Research Institute for Medical Sciences, Inha University Research and Business Foundation, Incheon, Korea; 5 Department of Surgery, lnha University Hospital, Incheon, Korea

**Keywords:** Cancer genetics, Thyroid cancer

## Abstract

Microgravity in space impacts human health. In particular, thyroid cancer, which has a high incidence rate, has been the subject of numerous studies with respect to microgravity. However, most studies have focused on Western follicular thyroid cancer cell lines, while data regarding the effects of microgravity on Asian cell lines are lacking. Therefore, we aimed to investigate the effect of simulated ground-based microgravity on two Korean thyroid cancer cell lines, namely SNU-790 and SNU-80. We found that both cell lines formed multicellular spheroids under simulated microgravity. Gene expression analysis revealed that in SNU-790 cells, histone-related genes were upregulated and microRNA-related genes were downregulated. Meanwhile, in SNU-80 cells, genes related to the cellular response to hypoxia were downregulated. These findings contribute to a better understanding of the effects of microgravity on thyroid cancer cells. Further validation studies and clinical significance analyses are needed to fully understand the implications of these findings.

## Introduction

Since the advent of space travel on April 12, 1961, extensive space exploration has been conducted^1^. The space environment is unique with microgravity and cosmic radiation, which cause damage and structural changes at the molecular and cellular levels, resulting in pathological conditions^2^. The effects of microgravity on various biological processes, including cell differentiation, proliferation, growth, apoptosis, adhesion, migration, invasion, and metastasis, have been studied^2–4^. In particular, cells exposed to microgravity form three-dimensional aggregates known as multicellular spheroids (MCSs) because of alterations in the cytoskeleton and extracellular matrix^1,3^.

Despite advancements in medical science, definitive strategies for cancer prevention and treatment remain elusive^5^. Numerous studies have reported the inhibitory effects of microgravity on the growth and viability of cancer cells^3–5^. As such, the microgravity environment holds potential as a novel therapeutic strategy for cancer^5^. In particular, owing to the steadily increasing incidence of thyroid cancer, research on its characteristics under microgravity environments has been actively conducted^6^. In fact, MCSs have been reported within normal thyroid cell and thyroid cancer cell cultures under microgravity^3,4,7,8^. Moreover, in thyroid cancer cell cultures under microgravity, the expression of B cell lymphoma-1 (BCL-2)—an antiapoptotic protein—and proinflammatory cytokines, including interleukin (IL)-6, IL-7, and IL-17, is upregulated^5,9^. Under zero gravity conditions, the cytoskeleton of thyroid cancer cells also undergoes disintegration, accompanied by upregulated vimentin expression and increased production of extracellular matrix proteins^10^.

However, thyroid cancer cells used in most zero gravity-related studies are follicular thyroid cancer (FTC) cells, whereas studies with papillary thyroid cancer (PTC) cells are rare^3,8,10^. Most microgravity studies using thyroid cells have been conducted using cells obtained from Westerns (Nthy-ori 3-1, HTU 5, FTC-133, UCLA RO82-W-1, and ONCO-DG1), while no such study exists using thyroid cells derived from Asians^11–15^. In 2007, Koh et al. established SNU-790 and SNU-80 cells derived from Korean breast cancer and papillary thyroid cancer, respectively. They reported on the characteristics of these cells and demonstrated that these characteristics were maintained even after numerous passages^16^. This study aims to characterize the Korean-derived thyroid cancer cells, SNU-790 and SNU80, cultured under simulated microgravity conditions^16^.

## Results

### MCS formation

**Fig. 1:**
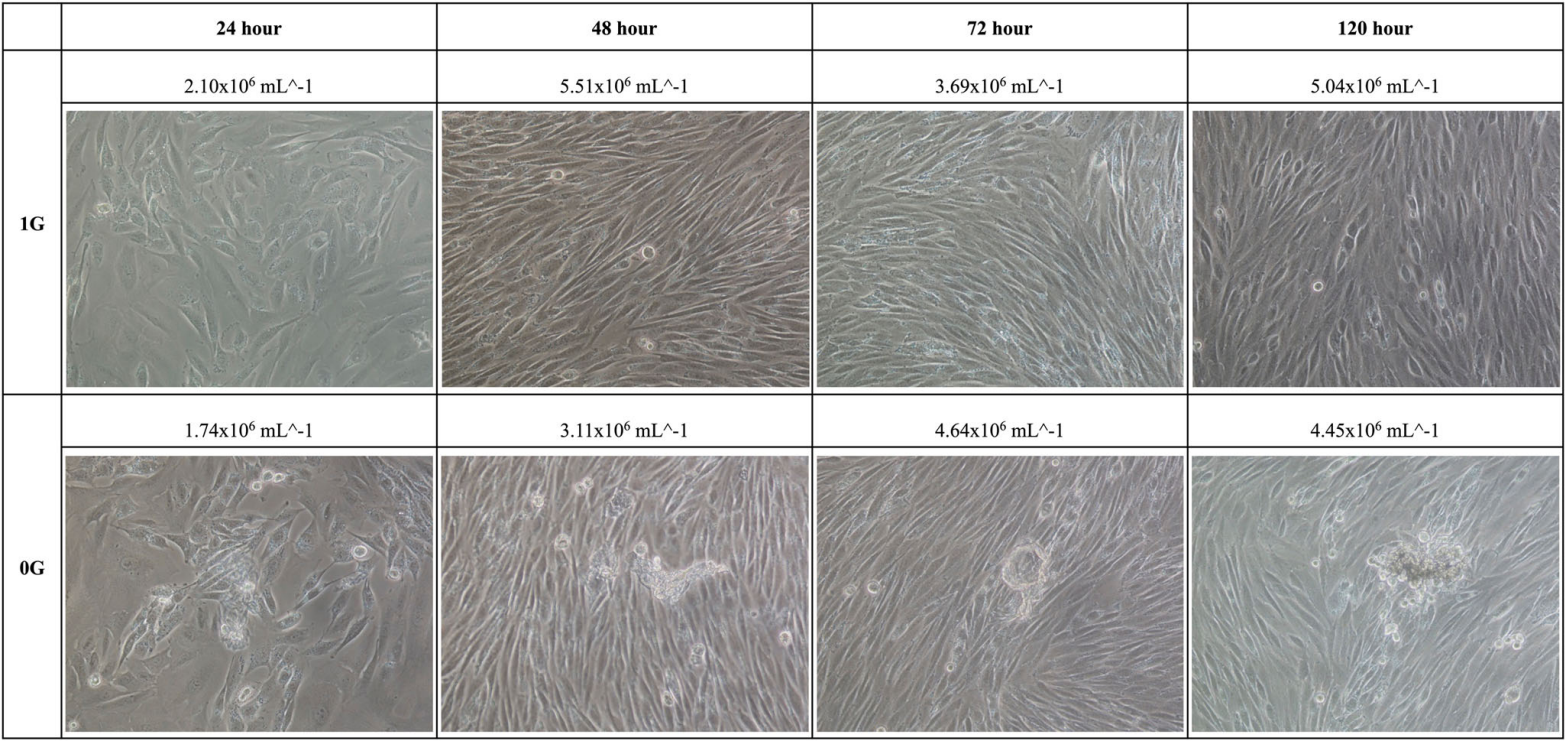
Phase-contrast microscopy images of SNU-790 under normal gravity and simulated microgravity conditions after 24 h, 48 h, 72 h, and 120 h of treatment. 1 G, normal gravity, 0 G simulated microgravity.

**Fig. 2:**
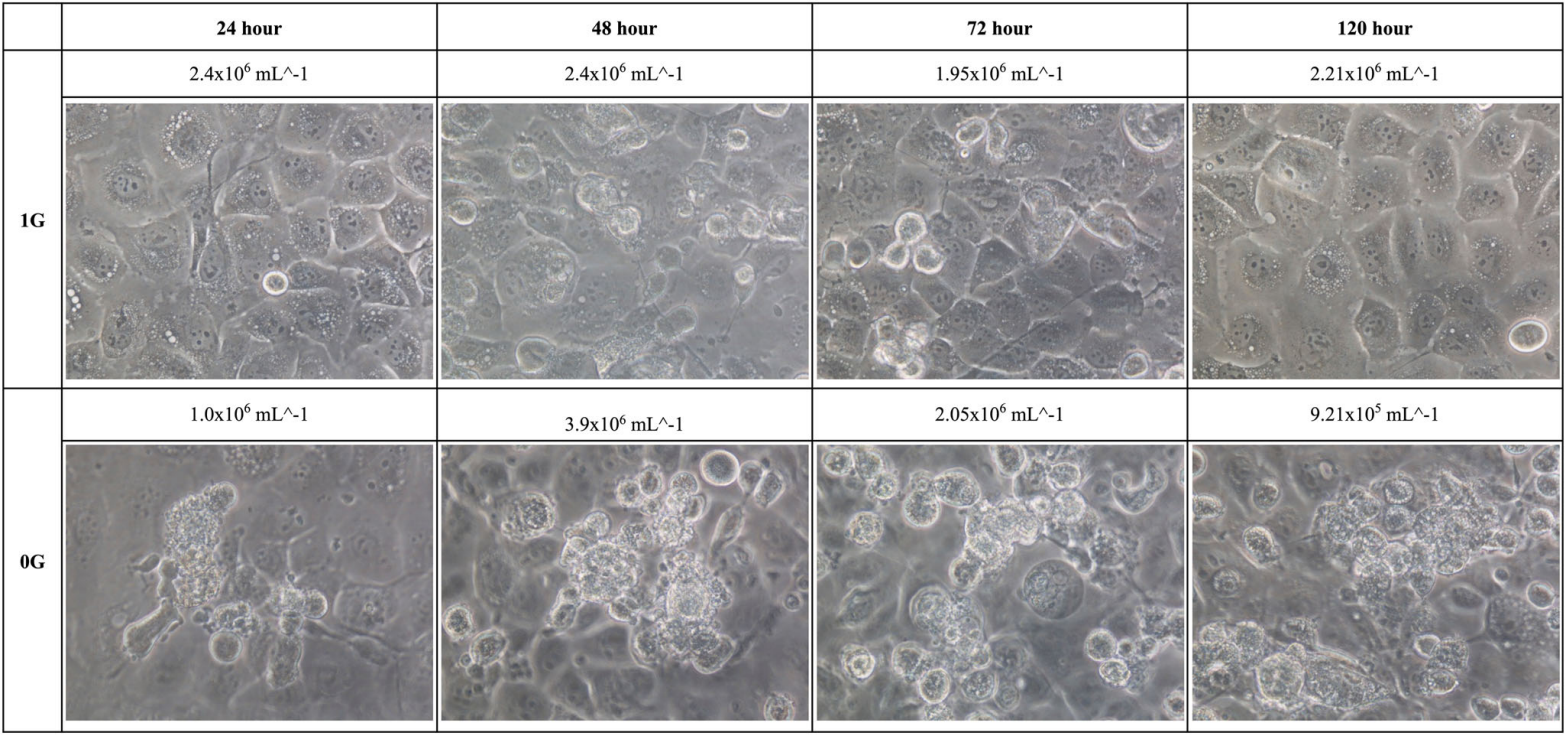
Phase-contrast microscopy images of SNU-80 under normal gravity and simulated microgravity conditions after 24 h, 48 h, 72 h, and 120 h of treatment. 1 G, normal gravity, 0 G simulated microgravity.

Figures 1 and 2 show changes over time in the morphology and number of SNU-790 and SNU-80 cells cultured under simulated microgravity and normal gravity (1 G) conditions. Adherent SNU-790 cells were identified as elongated spindle epithelial cells. Over time, adherent SNU-790 cells became densely cultured. SNU-790 cells began to form three-dimensional aggregates after 24 h of incubation under simulated microgravity. At 48 h and 72 h, slightly larger cellular aggregates were observed, and by 120 h, MCSs were discovered. In contrast, SNU-790 cells cultured under normal gravity conditions only displayed a two-dimensional structure with adherent cells. Meanwhile, adherent SNU-80 cells were observed as polygonal epithelial cells with large, round nuclei. These cells reached confluence over time. The formation of MCSs was observed in SNU-80 cells after 24-hour incubation under simulated microgravity, with the number of MCSs increasing over time. However, no MCSs were observed in SNU-80 cells cultured under 1 G.

### Altered gene expression in SNU-790 cells

**Fig. 3:**
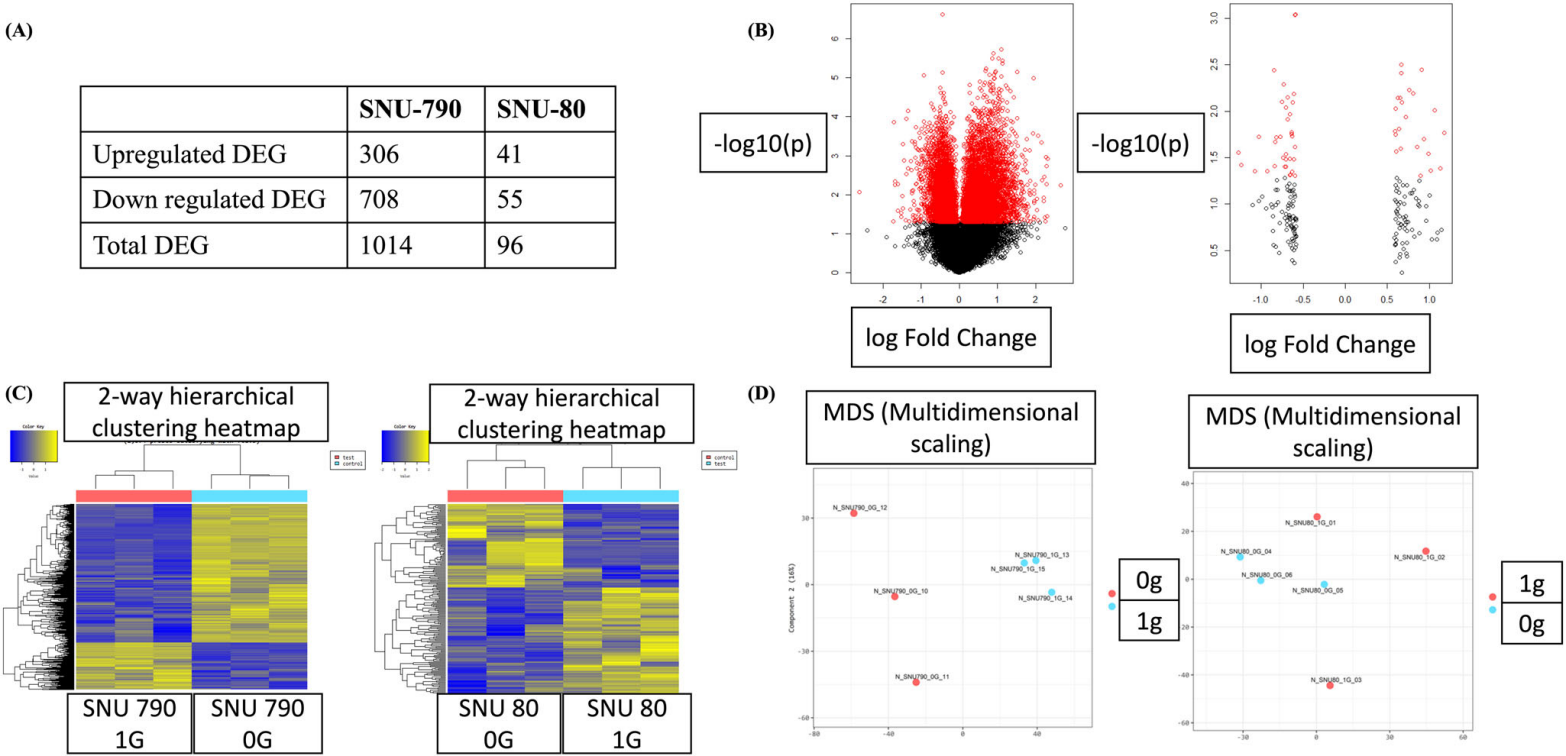
Analysis of differentially expressed genes in SNU-790 and SNU-80 cells under simulated microgravity conditions compared to those under normal gravity. (**A**) Number of differentially expressed genes; (**B**) volcano plot; (**C**) heatmap; (**D**) principal component analysis. In (**b**), (**c**), and (**d**), left panels represent SNU-790, right panels represent SNU-80.

Differentially expressed gene (DEG) analysis in SNU-790 cells revealed 1014 DEGs under simulated microgravity compared with those under 1 G conditions. Of these, 306 were upregulated and 708 were downregulated (Fig. 3). Moreover, heatmap and principal component analysis (PCA) results showed that SNU-790 cultured in simulated microgravity exhibited transcriptional profiles distinct from those of cells cultured under 1 G (Fig. 3).

**Fig. 4:**
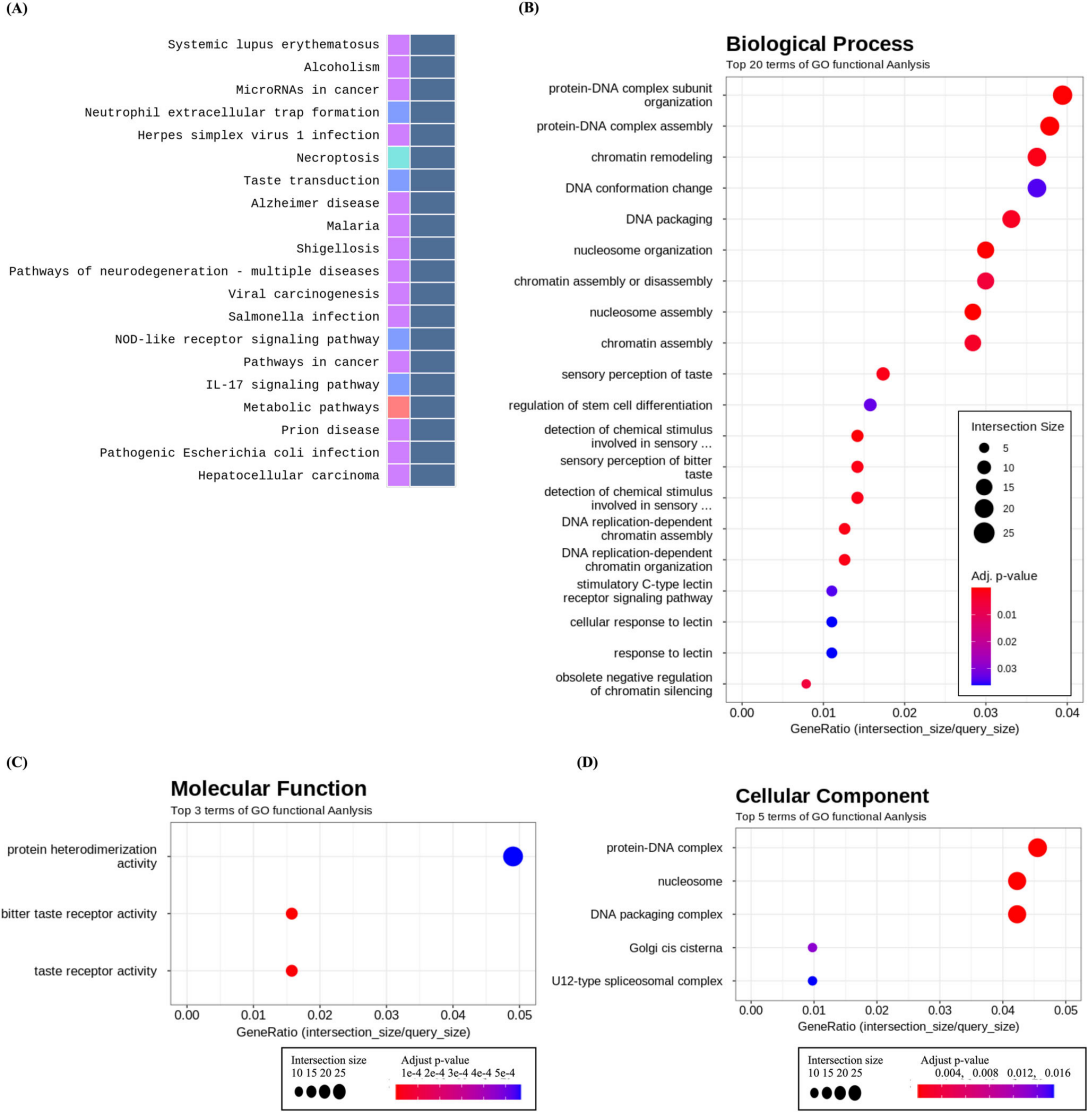
Functional annotation analysis of differentiallyexpressed genes in SNU-790 Cells under simulated microgravity conditions. **A** Top 20 KEGG Pathways Enrichment Analysis, **B** GeneOntology enrichment analysis of Biological Processes, **C** GeneOntology enrichment analysis of Molecular Function, and **D** GeneOntology enrichment analysis of Cellular Component.

Figure 4 and Table 1 summarize the results of KEGG pathway and Gene Ontology (GO) enrichment analyses conducted on SNU-790 cells cultured under simulated microgravity conditions. The DEGs identified in SNU-790 cells were associated with 44 KEGG pathways. Among the top 20 pathways analyzed, systemic lupus erythematosus (ID:05322), alcoholism (ID:05034), neutrophil extracellular trap formation (ID:04613), necroptosis (ID:04217), shigellosis (ID:05131), and viral carcinogenesis (ID:05203) were observed to express more histone-cluster-related genes than other genes. Moreover, a total of 32 GO biological process (GO:BP) terms, 3 GO molecular function (GO:MF) terms, and 5 GO cellular component (GO:CC) terms were enriched. Protein-DNA complex subunit organization (GO:BP, GO:0071824), chromatin remodeling (GO:BP, GO:0006338), protein heterodimerization activity (GO:MF, GO:0046982), and nucleosome (GO:CC, GO:0000786), as well as similar terms were related to the overexpression of genes related to the histone cluster. Meanwhile, analysis of DEGs in SNU-790 cells cultured under simulated microgravity conditions revealed an overexpression of genes associated with histone clusters: *HIST1H3G* (fold change [FC] 3.286, adjusted *P* value [adj. *P*] < 0.001), *HIST1H3B* (FC 3.066, adj. *P* < 0.001), *HISTH2AB* (FC 2.555, adj. *P* < 0.001), *HIST1H1B* (FC 2.382, adj. *P* < 0.001), *HIST1H2AI* (FC 2.140, adj. *P* < 0.001), and *HIST1H1E* (FC 2.072, adj. *P* < 0.001; Table 2).

**Table 1:** KEGG pathway analysis and Gene Ontology enrichment analysis of differentially expressed genes in SNU-790 cells cultured under simulated microgravity conditions.

No.	Gene symbol	Fold change	Adjusted *P* value	Gene description
(A) Top five KEGG pathways associated with histone-related genes				
(A-a) Systemic lupus erythematosus (ID 05322, FDR <0.001)				
1	*HIST1H3G*	3.286	<0.001	Histone cluster 1, h3g
2	*HIST1H3B*	3.066	<0.001	Histone cluster 1, h3b
3	*HIST2H2AB*	2.555	<0.001	Histone cluster 2, h2ab
4	*HIST1H2AI*	2.140	<0.001	Histone cluster 1, h2ai
5	*HIST1H2AM*	3.286	<0.001	Histone cluster 1, h2am
6	*HIST1H2BG*	−1.513	<0.001	Histone cluster 1, h2bg
7	*HIST1H2AK*	−1.615	<0.001	Histone cluster 1, h2ak
8	*HIST2H2BF*	−1.679	0.005	Histone cluster 2, h2bf
9	*C4A*	−2.095	<0.001	Complement component 4A (Rodgers blood group)
10	*C4B*	−2.130	<0.001	Complement component 4B (Chido blood group)
(A-b) Alcoholism (ID 05034, FDR <0.001)				
1	*HIST1H3G*	3.286	<0.001	Histone cluster 1, h3g
2	*HIST1H3B*	3.066	<0.001	Histone cluster 1, h3b
3	*HIST2H2AB*	2.555	<0.001	Histone cluster 2, h2ab
4	*HIST1H2AI*	2.140	<0.001	Histone cluster 1, h2ai
5	*HIST1H2AM*	1.981	0.003	Histone cluster 1, h2am
6	*HIST1H2BG*	−1.513	<0.001	Histone cluster 1, h2bg
7	*HIST1H2AK*	−1.615	<0.001	Histone cluster 1, h2ak
8	*HIST2H2BF*	−1.679	0.005	Histone cluster 2, h2bf
9	*GNAI3*	−2.572	<0.001	Guanine nucleotide-binding protein (G protein), alpha inhibiting activity polypeptide 3
(A-c) Neutrophil extracellular trap formation (ID 04613, FDR <0.001)				
1	*HIST1H3G*	3.286	<0.001	Histone cluster 1, h3g
2	*HIST1H3B*	3.066	<0.001	Histone cluster 1, h3b
3	*HIST2H2AB*	2.555	<0.001	Histone cluster 2, h2ab
4	*HIST1H2AI*	2.140	<0.001	Histone cluster 1, h2ai
5	*HIST1H2AM*	1.981	0.003	Histone cluster 1, h2am
6	*HIST1H2BG*	−1.513	<0.001	Histone cluster 1, h2bg
7	*HIST1H2AK*	−1.615	<0.001	Histone cluster 1, h2ak
8	*HIST2H2BF*	−1.679	0.005	Histone cluster 2, h2bf
9	*SELP*	−1.888	0.004	Selectin P (granule membrane protein 140 kda, antigen CD62)
(A-d) Necroptosis (ID:04217, FDR < 0.001)				
1	*HIST2H2AB*	2.555	0.000	Histone cluster 2, h2ab
2	*HIST1H2AI*	2.140	0.000	Histone cluster 1, h2ai
3	*HIST1H2AM*	1.981	0.003	Histone cluster 1, h2am
4	*FADD*	1.864	0.000	Fas (TNFRSF6)-associated via death domain
5	*IL33*	1.802	0.000	Interleukin 33
6	*TRAF5*	−1.524	0.002	TNF receptor-associated factor 5
7	*HIST1H2AK*	−1.615	0.000	Histone cluster 1, h2ak
(A-e) Shigellosis (ID:05131, FDR <0.001)				
1	*HIST1H3G*	3.286	0.000	Histone cluster 1, h3g
2	*HIST1H3B*	3.066	0.000	Histone cluster 1, h3b
3	*CXCL8*	2.417	0.000	Chemokine (C-X-C motif) ligand 8
4	*HIST2H3A*	1.809	0.000	Histone cluster 2, h3a
5	*HIST2H3A*	1.809	0.000	Histone cluster 2, h3a
6	*ACTR3B*	−1.516	0.009	ARP3 actin-related protein 3 homolog B (yeast)
7	*TRAF5*	−1.524	0.002	TNF receptor-associated factor 5
8	*NAIP*	−1.561	0.000	NLR family, apoptosis inhibitory protein
9	*SEPT11*	−1.705	0.000	Septin 11
10	*NAIP*	−1.766	0.000	NLR family, apoptosis inhibitory protein
(A-f) Viral carcinogenesis (ID:05203)				
1	*HIST1H4C*	1.819	0.000	Histone cluster 1, h4c
2	*HIST1H2BB*	1.787	0.011	Histone cluster 1, h2bb
3	*HIST1H4D*	1.729	0.000	Histone cluster 1, h4d
4	*CDK1*	1.638	0.000	Cyclin-dependent kinase 1
5	*HIST1H4K*	1.539	0.000	Histone cluster 1, h4k
6	*HIST1H2BG*	−1.513	0.000	Histone cluster 1, h2bg
7	*TRAF5*	−1.524	0.002	TNF receptor-associated factor 5
8	*HIST2H2BF*	−1.679	0.005	Histone cluster 2, h2bf
9	*GTF2H2*	−1.882	0.000	General transcription factor IIH subunit 2

(B) Top five KEGG pathways associated with microRNA-related genes				
(B-a) MicroRNAs in cancer (ID 05206, FDR< 0.001)				
1	*MIR520E*	2.017	0.001	MicroRNA 520e
2	*MIR146A*	1.610	<0.001	MicroRNA 146a
3	*MIRLET7C*	−2.101	<0.001	MicroRNA let-7c
4	*MIRLET7F1*	−2.151	0.001	MicroRNA let-7f-1
5	*ZEB2*	−2.281	<0.001	Zinc finger E-box binding homeobox 2
6	*MIR222*	−2.549	<0.001	MicroRNA 222
7	*MIRLET7A2*	−2.835	<0.001	MicroRNA let-7a-2

(C) Top five Gene Ontology enrichment terms associated with histone-related genes				
(C-a) Protein-DNA complex subunit organization (GO:BP, GO:0071824, adj. *P* <0.001)				
1	*HIST1H3G*	3.286	<0.001	Histone cluster 1, h3g
2	*HIST1H3B*	3.066	<0.001	Histone cluster 1, h3b
3	*HIST1H1B*	2.382	<0.001	Histone cluster 1, h1b
4	*HIST1H1E*	2.072	<0.001	Histone cluster 1, h1e
5	*HIST1H4C*	1.819	<0.001	Histone cluster 1, h4c
6	*HIST1H2BG*	−1.513	<0.001	Histone cluster 1, h2bg
7	*CHAF1B*	−1.633	0.005	Chromatin assembly factor 1, subunit B (p60)
8	*HIST2H2BF*	−1.679	0.005	Histone cluster 2, h2bf
9	*CHAF1B*	−1.749	0.040	Chromatin assembly factor 1, subunit B (p60)
(C-b) Protein-DNA complex assembly (GO:BP, GO:0065004, adj. *P* < 0.001)				
1	*HIST1H3G*	3.286	<0.001	Histone cluster 1, h3g
2	*HIST1H3B*	3.066	<0.001	Histone cluster 1, h3b
3	*HIST1H1B*	2.382	<0.001	Histone cluster 1, h1b
4	*HIST1H1E*	2.072	<0.001	Histone cluster 1, h1e
5	*HIST1H4C*	1.819	<0.001	Histone cluster 1, h4c
6	*HIST1H2BG*	−1.513	<0.001	Histone cluster 1, h2bg
7	*CHAF1B*	−1.633	0.005	Chromatin assembly factor 1, subunit B (p60)
8	*HIST2H2BF*	−1.679	0.005	Histone cluster 2, h2bf
9	*CHAF1B*	−1.749	0.040	Chromatin assembly factor 1, subunit B (p60)
(C-c) Chromatin remodeling (GO:BP, GO:0006338, adj. *P* = 0.002)				
1	*HIST1H3G*	3.286	<0.001	Histone cluster 1, h3g
2	*HIST1H3B*	3.066	<0.001	Histone cluster 1, h3b
3	*HIST1H1B*	2.382	<0.001	Histone cluster 1, h1b
4	*HIST1H1E*	2.072	<0.001	Histone cluster 1, h1e
5	*HIST1H4C*	1.819	<0.001	Histone cluster 1, h4c
6	*PABPC1L*	−1.627	0.001	Poly(A) binding protein, cytoplasmic 1-like
7	*CHAF1B*	−1.633	0.005	Chromatin assembly factor 1, subunit B (p60)
8	*HIST2H2BF*	−1.679	0.005	Histone cluster 2, h2bf
9	*CHAF1B*	−1.749	0.040	Chromatin assembly factor 1, subunit B (p60)
10	*KDM4C*	−1.889	0.001	Lysine (K)-specific demethylase 4C
(C-d) Protein heterodimerization activity (GO:MF, GO:0046982, adj. *P* = 0.001)				
1	*HIST1H3G*	3.286	0.000	Histone cluster 1, h3g
2	*HIST1H3B*	3.066	0.000	Histone cluster 1, h3b
3	*HIST2H2AB*	2.555	0.000	Histone cluster 2, h2ab
4	*HIST1H2AI*	2.140	0.000	Histone cluster 1, h2ai
5	*HIST1H2AM*	1.981	0.003	Histone cluster 1, h2am
6	*HIST1H2BG*	−1.513	0.000	Histone cluster 1, h2bg
7	*HIST1H2AK*	−1.615	0.000	Histone cluster 1, h2ak
8	*TPM1*	−1.623	0.000	Tropomyosin 1 (alpha)
9	*HIST2H2BF*	−1.679	0.005	Histone cluster 2, h2bf
10	*TENM4*	−2.150	0.000	Teneurin transmembrane protein 4
(C-e) Nucleosome (GO:CC, GO:0000786, adj. *P* < 0.001)				
1	*HIST1H3G*	3.286	0.000	Histone cluster 1, h3g
2	*HIST1H3B*	3.066	0.000	Histone cluster 1, h3b
3	*HIST2H2AB*	2.555	0.000	Histone cluster 2, h2ab
4	*HIST1H1B*	2.382	0.000	Histone cluster 1, h1b
5	*HIST1H2AI*	2.140	0.000	Histone cluster 1, h2ai
6	*HIST1H2BG*	−1.513	0.000	Histone cluster 1, h2bg
7	*HIST1H2AK*	−1.615	0.000	Histone cluster 1, h2ak
8	*HIST2H2BF*	−1.679	0.005	Histone cluster 2, h2bf

(D) Top five Gene Ontology enrichment terms associated with microRNA-related genes				
(D-a) Gene silencing by miRNA (GO:BP, GO:0035195, adj. *P* < 0.001)				
1	*MIR520E*	2.017	0.001	MicroRNA 520e
2	*MIR519A2*	1.905	0.022	MicroRNA 519a-2
3	*MIR196A1*	1.868	0.023	MicroRNA 196a-1
4	*MIR328*	1.616	0.003	MicroRNA 328
5	*MIR146A*	1.610	0.000	MicroRNA 146a
6	*NEAT1*	−2.311	0.000	Nuclear paraspeckle assembly transcript 1 (non-protein coding)
7	*MIR573*	−2.359	0.000	MicroRNA 573
8	*MIR604*	−2.396	0.000	MicroRNA 604
9	*MIR222*	−2.549	0.000	MicroRNA 222
10	*MIRLET7A2*	−2.835	0.000	MicroRNA let-7a-2
(D-b) Posttranscriptional gene silencing (GO:BP, GO:0035194, adj. *P* < 0.001)				
1	*MIR520E*	2.017	0.001	MicroRNA 520e
2	*MIR519A2*	1.905	0.022	MicroRNA 519a-2
3	*MIR196A1*	1.868	0.023	MicroRNA 196a-1
4	*MIR328*	1.616	0.003	MicroRNA 328
5	*MIR146A*	1.610	0.000	MicroRNA 146a
6	*NEAT1*	−2.311	0.000	Nuclear paraspeckle assembly transcript 1 (non-protein coding)
7	*MIR573*	−2.359	0.000	MicroRNA 573
8	*MIR604*	−2.396	0.000	MicroRNA 604
9	*MIR222*	−2.549	0.000	MicroRNA 222
10	*MIRLET7A2*	−2.835	0.000	MicroRNA let-7a-2

*KEGG* Kyoto Encyclopedia of Genes and Genomes, *FDR* false discovery rate, *No* number, *GO* gene ontology, *BP* biological process, *adj.P* adjusted *P* value, *MF* molecular function, *CC* cellular component.

(Gene names are to be presented in italics).

**Table 2:** Comparative analysis of differentially expressed genes in SNU-790 cells cultured under simulated microgravity and normal gravity conditions.

No.	Gene symbol	Fold change	Adjusted *P* value	Gene description
(A) Top 20 upregulated genes in SNU-790 cells in simulated microgravity				
1	*SNORA14A*	6.173	<0.001	Small nucleolar RNA, H/ACA box 14A
2	*HIST1H3G*	3.286	<0.001	Histone cluster 1, h3g
3	*TNFAIP6*	3.223	<0.001	Tumor necrosis factor, alpha-induced protein 6
4	*HIST1H3B*	3.066	<0.001	Histone cluster 1, h3b
5	*SNORA11*	2.734	<0.001	Small nucleolar RNA, H/ACA box 11
6	*HIST2H2AB*	2.555	<0.001	Histone cluster 2, h2ab
7	*CXCL8*	2.417	<0.001	Chemokine (C-X-C motif) ligand 8
8	*HIST1H1B*	2.382	<0.001	Histone cluster 1, h1b
9	*METRN*	2.230	<0.001	Meteorin, glial cell differentiation regulator
10	*DNLZ*	2.217	<0.001	DNL-type zinc finger
11	*SNORA38B*	2.211	<0.001	Small nucleolar RNA, H/ACA box 38B
12	*C1QTNF1*	2.201	<0.001	C1q and tumor necrosis factor related protein 1
13	*RNU5F-1*	2.175	<0.001	RNA, U5F small nuclear 1
14	*MYDGF*	2.152	<0.001	Myeloid-derived growth factor
15	*HIST1H2AI*	2.140	<0.001	Histone cluster 1, h2ai
16	*SERPINB2*	2.091	<0.001	Serpin peptidase inhibitor, clade B (ovalbumin), member 2
17	*TRAJ44*	2.078	0.002	T cell receptor alpha joining 44
18	*HIST1H1E*	2.072	<0.001	Histone cluster 1, h1e
19	*TUBA3C*	2.037	<0.001	Tubulin, alpha 3c
20	*MT1JP*	2.025	0.010	Metallothionein 1J, pseudogene

(B) Top 20 downregulated genes in SNU-790 cells in simulated microgravity				
21	*MIR4798*	−3.437	<0.001	MicroRNA 4798
22	*MIR548K*	−3.234	<0.001	MicroRNA 548k
23	*MIR4762*	−3.217	<0.001	MicroRNA 4762
24	*MIR548O2*	−3.111	<0.001	MicroRNA 548o-2
25	*PCDHB13*	−3.098	<0.001	Protocadherin beta 13
26	*MIR1284*	−3.066	<0.001	MicroRNA 1284
27	*MIR4524A*	−2.925	<0.001	MicroRNA 4524a
28	*MIRLET7A2*	−2.835	<0.001	MicroRNA let-7a-2
29	*SNORA31*	−2.790	<0.001	Small nucleolar RNA, H/ACA box 31
30	*IFNE*	−2.779	<0.001	Interferon, epsilon
31	*PWAR6*	−2.759	<0.001	Prader Willi/Angelman region RNA 6
32	*MIR4668*	−2.672	<0.001	MicroRNA 4668
33	*SNORA16B*	−2.656	<0.001	Small nucleolar RNA, H/ACA box 16B
34	*TBC1D3E*	−2.650	<0.001	TBC1 domain family, member 3E
35	*RPS15AP10*	−2.620	<0.001	Ribosomal protein S15a pseudogene 10
36	*MIR4742*	−2.613	<0.001	MicroRNA 4742
37	*MIR4441*	−2.607	<0.001	MicroRNA 4441
38	*MIR4500*	−2.597	<0.001	MicroRNA 4500
39	*GLIS3*	−2.589	<0.001	GLIS family zinc finger 3
40	*GNAI3*	−2.572	<0.001	Guanine nucleotide binding protein (G protein), alpha inhibiting activity polypeptide 3

(Gene names are to be presented in italics).

KEGG pathway enrichment analysis of SNU-790 cells under simulated microgravity revealed that most genes related to cancer-associated microRNAs (ID:05206) were downregulated. Additionally, terms related to gene silencing, such as gene silencing by miRNA (GO:BP, GO:0035195) and posttranscriptional gene silencing (GO:BP, GO:0035194), were associated with low expression of microRNA-related genes. The top genes downregulated in the simulated microgravity culture of SNU-790 were MIR4798 (FC − 3.437, adj. *P* < 0.001), MIR548K (FC − 3.234, adj. *P* < 0.001), MIR4762 (FC − 3.217, adj. *P* < 0.001), MIR548O2 (FC − 3.111, adj. *P* < 0.001), MIR1284 (FC − 3.066, adj. *P* < 0.001), MIR4524A (FC − 2.925, adj. *P* < 0.001), MIRLET7A2 (FC − 2.835, adj. *P* < 0.001), MIR4668 (FC − 2.672, adj*. P* < 0.001), MIR4742 (FC -2.613, adj*. P* < 0.001), MIR4441 (FC -2.607, adj*. P* < 0.001), and MIR4500 (FC -2.597, adj*. P* < 0.001).

### Altered gene expression in SNU-80 cells

SNU-80 cell analysis revealed 96 DEGs under simulated microgravity compared with those under 1 G. Of these, 41 were upregulated and 55 were downregulated (Fig. 3). The heatmap and PCA showed that SNU-80 cultured in simulated microgravity had transcriptional profiles distinct from those of cells cultured in 1 G (Fig. 3).

**Fig. 5:**
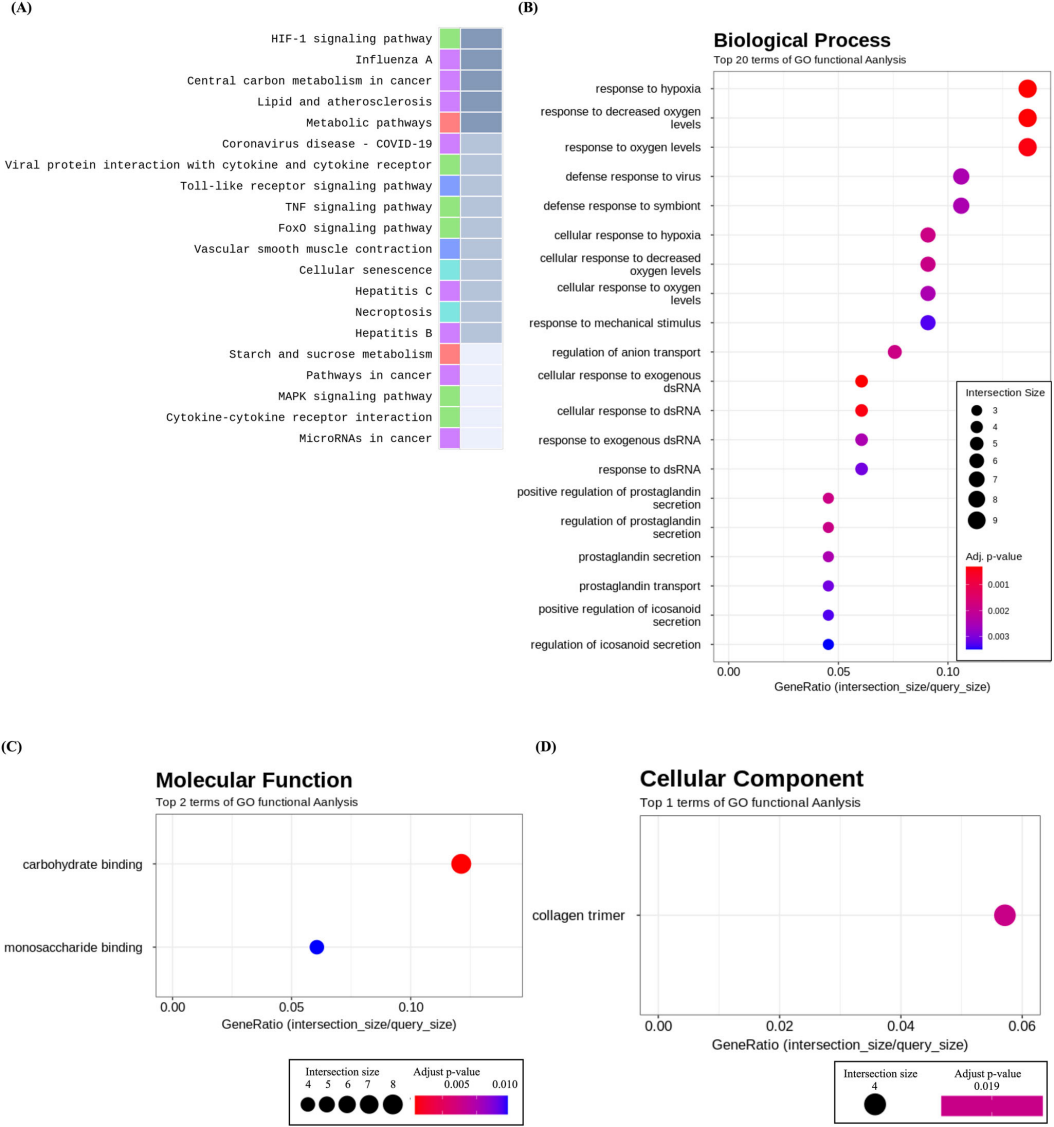
Functional annotation analysis of differentially expressed genes in SNU-80 Cells under simulated microgravity conditions. **A** Top 20 KEGG Pathways Enrichment Analysis, **B** Gene Ontology enrichment analysis of Biological Processes, **C** Gene Ontology enrichment analysis of Molecular Function, and **D** Gene Ontology enrichment analysis of Cellular Component.

Figure 5 and Table 3 summarize the results of the KEGG pathway enrichment and GO enrichment analyses of SNU-80 cells under simulated microgravity. A total of 15 KEGG pathways showed remarkable results in the enrichment analysis using the SNU-80 DEG set. The HIF-1 signaling pathway (ID 04066, *P* = 0.001, FDR = 0.159) was analyzed, and genes related to response to hypoxic conditions, including *EDN1* (FC − 1.979, adj. *P* < 0.001), *PDK1* (FC − 1.844, adj. *P* < 0.001), *ENO2* (FC − 1.628, adj. *P* < 0.001), and *HK2* (FC − 1.552, adj. *P* < 0.001), were downregulated. Moreover, DEGs related to the response to hypoxia were observed in pathways such as central carbon metabolism in cancer (ID 05230, *P* = 0.008, adj. *P* = 0.217), lipid and atherosclerosis (ID 05417, *P* = 0.008, adj. *P* = 0.217), and metabolic pathways (ID 01100, *P* = 0.008, adj. *P* = 0.217). A total of 164 GO:BP terms, 3 GO molecular function (GO:MF) terms, and 5 GO cellular component (GO:CC) terms were analyzed. A total of six GO:BP terms were associated with the response to hypoxic conditions, including response to hypoxia (GO:BP, GO:0001666, adj. *P* < 0.001), response to decreased oxygen levels (GO:BP, GO:0036293, adj. *P* < 0.001), and response to oxygen levels (GO:BP, GO:0070482, adj. *P* < 0.001).

**Table 3:** KEGG pathway analysis and Gene Ontology enrichment analysis of differentially expressed genes in SNU-80 cells cultured under simulated microgravity conditions.

No	Gene symbol	Fold change	Adjusted *P* value	Gene description
(A) Top five downregulated KEGG pathways associated with genes related to response to hypoxia				
(A-a) HIF-1 signaling pathway (ID 04066, *P* = 0.001, adj. *P* = 0.159)				
1	*HK2*	−1.552	<0.001	Hexokinase 2
2	*ENO2*	−1.628	<0.001	Enolase 2 (gamma, neuronal)
3	*PDK1*	−1.844	<0.001	Pyruvate dehydrogenase kinase, isozyme 1
4	*EDN1*	−1.979	<0.001	Endothelin 1
(A-b) Central carbon metabolism in cancer (ID 05230, *P* = 0.008, adj. *P* = 0.217)				
1	*HK2*	−1.552	<0.001	Hexokinase 2
2	*PDK1*	−1.844	<0.001	Pyruvate dehydrogenase kinase, isozyme 1
(A-c) Lipid and atherosclerosis (ID 05417, *P* = 0.008, adj. *P* = 0.217)				
1	*VLDLR*	−1.792	<0.001	Very low density lipoprotein receptor
(A-d) Metabolic pathways (ID 01100, *P* = 0.008, adj. *P* = 0.217)				
1	*CPOX*	2.114	0.009	Coproporphyrinogen oxidase
2	*GBE1*	−1.536	<0.001	Glucan (1,4-alpha-), branching enzyme 1
3	*HK2*	−1.552	<0.001	Hexokinase 2
4	*ENO2*	−1.628	<0.001	Enolase 2 (gamma, neuronal)
5	*P4HA1*	−1.653	<0.001	Prolyl 4-hydroxylase, alpha polypeptide I
6	*CA9*	−2.211	<0.001	Carbonic anhydrase IX

(B) Top five Gene Ontology terms associated with genes related to the response to hypoxia				
(B-a) Response to hypoxia (GO:BP, GO:0001666, adj. *P* < 0.001)				
1	*NDRG1*	−1.677	<0.001	*N-myc* downstream regulated 1
2	*PDK1*	−1.844	<0.001	Pyruvate dehydrogenase kinase, isozyme 1
3	*EDN1*	−1.979	<0.001	Endothelin 1
4	*BNIP3*	−2.130	<0.001	*BCL2*/adenovirus *E1B* 19 kDa interacting protein 3
5	*CA9*	−2.211	<0.001	Carbonic anhydrase IX
(B-b) Response to decreased oxygen levels (GO:BP, GO:0036293, adj. *P* < 0.001)				
1	*NDRG1*	−1.677	<0.001	*N-myc* downstream regulated 1
2	*PDK1*	−1.844	<0.001	Pyruvate dehydrogenase kinase, isozyme 1
3	*EDN1*	−1.979	<0.001	Endothelin 1
4	*BNIP3*	−2.130	<0.001	*BCL2*/adenovirus *E1B* 19 kDa interacting protein 3
5	*CA9*	−2.211	<0.001	Carbonic anhydrase IX
(B-c) Response to oxygen levels (GO:BP, GO:0070482, adj. P < 0.001)				
1	*NDRG1*	−1.677	<0.001	*N-myc* downstream regulated 1
2	*PDK1*	−1.844	<0.001	Pyruvate dehydrogenase kinase, isozyme 1
3	*EDN1*	−1.979	<0.001	Endothelin 1
4	*BNIP3*	−2.130	<0.001	*BCL2*/adenovirus *E1B* 19 kDa interacting protein 3
5	*CA9*	−2.211	<0.001	Carbonic anhydrase IX
(B-d) Cellular response to hypoxia (GO:BP, GO:0071456, adj. *P* = 0.002)				
1	*STC1*	−1.524	<0.001	Stanniocalcin 1
2	*NDRG1*	−1.677	<0.001	*N-myc* downstream regulated 1
3	*PDK1*	−1.844	<0.001	Pyruvate dehydrogenase kinase, isozyme 1
4	*EDN1*	−1.979	<0.001	Endothelin 1
5	*BNIP3*	−2.130	<0.001	BCL2/adenovirus E1B 19 kDa interacting protein 3
(B-e) Cellular response to decreased oxygen levels (GO:BP, GO:0036294, adj. P = 0.002)				
1	*STC1*	−1.524	<0.001	Stanniocalcin 1
2	*NDRG1*	−1.677	<0.001	*N-myc* downstream regulated 1
3	*PDK1*	−1.844	<0.001	Pyruvate dehydrogenase kinase, isozyme 1
4	*EDN1*	−1.979	<0.001	Endothelin 1
5	*BNIP3*	−2.130	<0.001	*BCL2*/adenovirus *E1B* 19 kDa interacting protein 3

(Gene names are to be presented in italics).

Table 4 summarizes the top 17 up- and 20 downregulated mRNAs from microarray analysis of SNU-80 cells in simulated microgravity culture. *CA9* (FC − 2.211, adj. *P* < 0.001), *BNIP3* (FC − 2.130, adj. *P* < 0.001), *NPPB* (FC − 2.017, adj. *P* < 0.001), *EDN1* (FC − 1.979, adj. *P* < 0.001), *PDK1* (FC − 1.844, adj. *P* < 0.001), *VLDLR* (FC − 1.792, adj. *P* < 0.001), and *NDRG1* (FC − 1.677, adj. *P* < 0.001) were downregulated under simulated microgravity in SNU-80 cells, and were related to the cellular response to hypoxia.

**Table 4:** Comparative analysis of differentially expressed genes in SNU-80 cells cultured under simulated microgravity and normal gravity conditions.

No.	Gene symbol	Fold change	Adjusted *P*-value	Gene description
(A) Top 17 upregulated genes in SNU-80 cells in simulated microgravity				
1	*MIR29A*	2.418	0.018	MicroRNA 29a
2	*CPOX*	2.114	0.009	Coproporphyrinogen oxidase
3	*GBP4*	1.902	<0.001	Guanylate binding protein 4
4	*TLR3*	1.797	0.039	Toll-like receptor 3
5	*IFI44L*	1.785	<0.001	Interferon-induced protein 44-like
6	*SERPINB4*	1.707	0.024	Serpin peptidase inhibitor, clade B (ovalbumin), member 4
7	*IFIT1*	1.645	<0.001	Interferon-induced protein with tetratricopeptide repeats 1
8	*GBP1*	1.629	0.024	Guanylate binding protein 1, interferon-inducible
9	*MIR4668*	1.600	<0.001	MicroRNA 4668
10	*IFIH1*	1.577	0.009	Interferon induced, with helicase C domain 1
11	*MIR4295*	1.573	0.011	MicroRNA 4295
12	*EVI2A*	1.570	0.039	Ecotropic viral integration site 2A
13	*IL24*	1.549	<0.001	Interleukin 24
14	*HERC6*	1.532	0.002	HECT and RLD domain containing E3 ubiquitin protein ligase family member 6
15	*XAF1*	1.516	0.001	XIAP associated factor 1
16	*RAB27B*	1.507	0.013	*RAB27B*, member *RAS* oncogene family
17	*STEAP1*	1.503	0.021	Six transmembrane epithelial antigen of the prostate 1

(B) Top 17 downregulated genes in SNU-80 cells in simulated microgravity				
21	*CA9*	−2.211	<0.001	Carbonic anhydrase IX
22	*NRN1*	−2.137	<0.001	Neuritin 1
23	*BNIP3*	−2.130	<0.001	*BCL2*/adenovirus *E1B* 19 kDa interacting protein 3
24	*OR10V2P*	−2.083	<0.001	Olfactory receptor, family 10, subfamily V, member 2 pseudogene
25	*FGF11*	−2.039	<0.001	Fibroblast growth factor 11
26	*NPPB*	−2.017	<0.001	Natriuretic peptide B
27	*EDN1*	−1.979	<0.001	Endothelin 1
28	*TMEM45A*	−1.944	<0.001	Transmembrane protein 45A
29	*LOX*	−1.910	<0.001	Lysyl oxidase
30	*LIMCH1*	−1.875	<0.001	LIM and calponin homology domains 1
31	*PDK1*	−1.844	<0.001	Pyruvate dehydrogenase kinase, isozyme 1
32	*MAMDC2*	−1.842	0.001	MAM domain containing 2
33	*VLDLR*	−1.792	<0.001	Very low density lipoprotein receptor
34	*UPK1A*	−1.756	0.004	Uroplakin 1A
35	*LOC154761*	−1.735	0.001	Family with sequence similarity 115, member C pseudogene
36	*NDRG1*	−1.677	<0.001	*N-myc* downstream regulated 1
37	*COL11A1*	−1.655	<0.001	Collagen, type XI, alpha 1
38	*P4HA1*	−1.653	<0.001	Prolyl 4-hydroxylase, alpha polypeptide I
39	*ENO2*	−1.628	<0.001	Enolase 2 (gamma, neuronal)
40	*SPAG4*	−1.571	<0.001	Sperm-associated antigen 4

(Gene names are to be presented in italics).

## Discussion

In this study, we investigated the morphological and gene expression differences between Korean-derived thyroid cancer cell lines, SNU-790 and SNU-80, cultured under normal gravity and simulated ground-based microgravity conditions. Our findings confirmed that SNU-790 and SNU-80 cells formed three-dimensional MCSs when cultured under simulated microgravity. Specifically, SNU-790 cells exhibited an upregulation of histone-related genes and downregulation of microRNA-related genes. Meanwhile, in SNU-80 cells genes associated with hypoxia response were downregulated. Collectively, this study provides valuable insights into the molecular and transcriptional changes of Korean thyroid cancer cells under simulated microgravity conditions, representing the first research using a Korean-derived cell line.

Research on cancer cells in a microgravity environment represents a highly promising field with the potential to enhance cancer prevention and treatment strategies^5^. The global incidence of thyroid cancer has been steadily increasing, particularly in Asian nations^17^. However, most studies investigating thyroid cancer cells under microgravity have predominantly focused on Western populations and utilized FTC cells^3,8,10^. This approach may not adequately capture the diverse characteristics of thyroid cancer. Hence, there is a need for research that specifically assesses thyroid cancer cells across different racial backgrounds to facilitate the development of more effective methods for preventing and treating thyroid cancer, while advancing our understanding of thyroid cancer treatment on a global scale.

In our study, we observed the formation of MCSs when SNU-790 and SNU-80 cells were cultured under simulated microgravity conditions. Unlike their adherent growth in normal gravity, these cells exhibited three-dimensional growth patterns under simulated microgravity, which has been observed previously^18^. MCS formation is not influenced by the cellular phenotype (normal or malignant) and is formed under microgravity regardless of cell type^9,18,19^. As such, culturing human FTC cells (UCLA RO82-W-1 cell line) under microgravity induces distinct characteristics, including the development of stress fibers and cellular extensions (lamellipodia, filopodia, and microvilli), compared to cells cultured under normal gravity conditions^20^. MCSs not only exhibit distinct structural characteristics but also mimic the behaviors of physiological processes in the human body^3^. Consequently, MCSs have emerged as valuable models for studying cancer metastasis and exploring drug-targeting strategies^3,18^. The confirmation of MCS formation in SNU-790 and SNU-80 cell lines represents a significant finding, offering a potential foundation for future investigations into the underlying mechanisms and the development of innovative treatment approaches utilizing non-Western thyroid cancer cell lines. However, the DEG, KEGG pathway enrichment, and GO analyses in the current study did not provide sufficient data to infer the mechanisms underlying MCS formation. Therefore, further systematic research in this regard is warranted.

Through this study, we found that the characteristics of SNU-790 and SNU-80 under simulated microgravity conditions were comparable to those of FTC cell lines derived from Western populations. In particular, we observed DEGs related to histone and microRNAs in SNU-790 cells. The involvement of histone and microRNAs in gene transcription suggests their potential impact on various cellular biological processes, including proliferation, growth, or differentiation, aligning with findings from previous research^2–4^. Additionally, a separate study reported the differential expression of over 100 microRNAs when thyroid cancer cells (FTC-133) were cultured at the International Space Station, indicating the potential for further investigations in this area^21^. However, it is important to note that our study focused on analyzing DEGs between simulated microgravity and normal gravity conditions, which limits our interpretation to correlation rather than establishing causal relationships.

SNU-790 cells cultured under simulated microgravity showed that histone-related genes were upregulated. Histones form nucleosomes with DNA, compose DNA-histone complexes called chromatin, and regulate RNA expression. Histones play a role in development and cancer growth through epigenetic changes, such as methylation, acetylation, and phosphorylation^22^. These findings align with the results of Singh et al., who observed that microgravity triggers epigenetic changes in human lymphocytes, including alterations in the expression of *DNMT1* and *HDAC*, which subsequently impacts gene expression^23^. Histone acetylation activates transcription by making DNA more accessible, and histone methylation regulates transcription by recruiting other transcription-related proteins^24^. The diversity of cellular functions related to histone modification emphasizes the need for additional research to provide more precise interpretation of our findings.

SNU-790 cells tended to under-express microRNA-related genes under simulated microgravity. MicroRNAs play a crucial role in regulating gene expression at the post-transcriptional level through RNA silencing^25^. They are involved in regulating various biological processes, such as cell differentiation, proliferation, apoptosis, energy metabolism, and cytokinesis^26^. Our findings align with previous studies that have reported the impact of weightlessness on the regulation of microRNA expression in colorectal cancer cells and leukocytes, leading to alterations in various biological processes associated with cancer^27,28^. However, in cancer cells, microRNAs can either induce or suppress tumor growth depending on the specific tissue and microRNA involved^25,26^. The precise implications of our findings on cancer characteristics remain unclear, and further analysis is needed to determine whether the observed under-expression of microRNAs affects oncogene or tumor suppressor gene expression.

In the case of SNU-80, the HIF-1 signaling pathway or GO terms related to hypoxia and under-expression of related genes were analyzed. HIF-1 plays a role in regulating oxygen homeostasis in the body. However, even when intratumoral hypoxia occurs because of the growth of cancer cells, it impacts vascularization and metabolic reprogramming for oxygen homeostasis, affecting cancer progression, invasion, and metastasis^29^. We conducted technical replicate experiments at least 10 times to eliminate confounding variables other than gravitational conditions and reduce experimental errors. After confirming the consistency of the results, we proceeded with the cDNA array analysis. Subsequently, we performed microarray analysis, confirming outcomes consistent across repetitions. As a result, our research team concluded that the effects observed were unlikely to be attributed to hypoxic conditions. Moreover, when comparing our findings to Xiao’s study, where FTC-133 cells were cultivated in a microgravity simulator, we noted similar enrichments of Gene Ontology (GO) terms related to the response to oxygen levels. This strengthens our inference that the observed effects in our study are likely associated with cancer-related pathways^30^. *CA9* was under-expressed in our study and reportedly causes angiogenesis and metabolic remodeling under conditions of hypoxia and acidosis while promoting invasiveness and metastatic propensity^31^. *HK2* is an enzyme that plays an important role in glycolysis. *HK2* overexpression has been reported in several carcinomas, whereas its deletion reportedly reduces cancer cell proliferation^32^. In addition, *PDK1* was also under-expressed in the present study. *PDK1* overexpression, which has been reported in melanoma, leukemia, and gastric carcinoma, results in poor overall survival or recurrence rates^33^. We observed differences in DEGs related to hypoxia in SNU-80 under microgravity, suggesting, in line with previous studies, that cancer cells in microgravity exhibit a less aggressive phenotype^3^.

The present study has a limitation in that it can only establish an association between gravitational conditions and DEGs. Additional research is needed to establish a causal relationship and to investigate the relations between DEG differences and specific clinical characteristics. Furthermore, the results obtained in this study necessitate well-designed research for validation.

In conclusion, this is the first study in which Korean thyroid cancer cell lines were used to study the effect of simulated microgravity on morphology and gene expression. SNU-790 and SNU-80 cultured under simulated microgravity formed MCSs. SNU-790 cells under microgravity revealed upregulation of histone-related genes and down-regulation of microRNA-related genes, whereas SNU-80 showed down-regulation of genes related to hypoxia responses. Our research demonstrated the potential of conducting microgravity studies using Korean thyroid cells. Additional research is needed to elucidate the mechanism and causal relationship in SNU-790 and SNU-80 cells exposed to simulated microgravity.

## Methods

### Cells and culture conditions

Human Korean PTC cells (SNU-790) and human anaplastic thyroid cancer cells (SNU-80) were purchased from the Korean cell line bank (https://www.cellosaurus.org; Seoul, Korea). Additional information regarding the acquisition of the cells used in this study can be obtained from the Seoul National University Cancer Research Institute. (https://cri.snu.ac.kr). The SNU-790 cell line used in this research was at passage number 10, while the SNU-80 cell line was at passage 8. The SNU-790 cell line (CVCL_5093) is a thyroid gland papillary carcinoma cell line obtained from the thyroid gland of a 72-year-old Korean male (NCBI Taxonomy: 9606). This cell line harbors *BRAF* (c.1799T>A) mutation, and its doubling time is 33 h. The SNU-80 cell line (CVCL_5097) is a thyroid gland anaplastic carcinoma cell line obtained from the thyroid gland of a 59-year-old Korean woman (NCBI Taxonomy: 9606) and harbors *BRAF* (c.1405 G > C) and *TP53* (c.832 C > G) mutations; its doubling time is 37 h.

Both cell lines were cultured in RPMI-1640 (Cytiva, Marlborough, MA, USA), supplemented with 10% fetal bovine serum (FBS; Cytiva), 1% penicillin-streptomycin (Thermo Fisher scientific, Waltham, MA, USA) in a humidified atmosphere of 5% CO_2_ at 37 °C. To proceed with cell culture experiments in simulated microgravity, 10^6^ cells were seeded in T-25 culture flasks (Corning, Corning, NY, USA).

### Simulated ground-based microgravity platform and cell culture

**Fig. 6:**
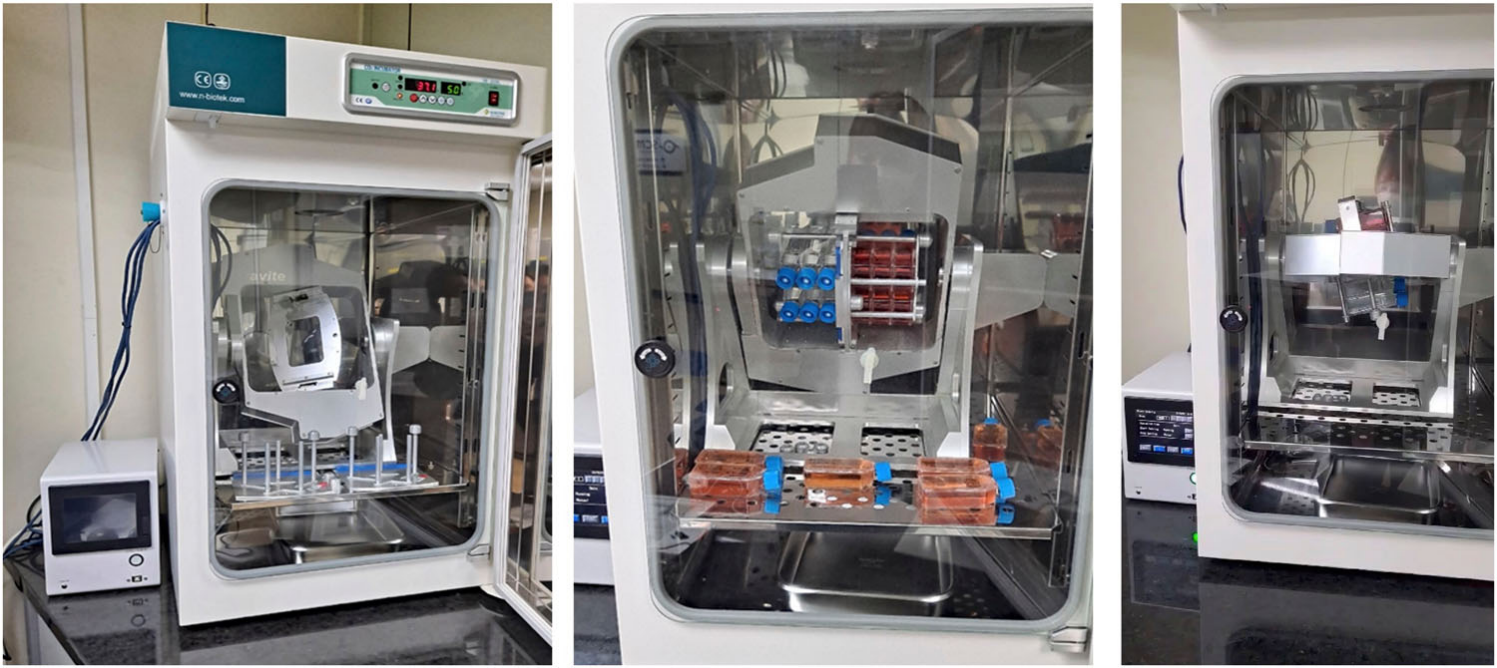
Simulator of microgravity, Gravity controller: “Gravite”. Main unit size (width × depth × height), 425 × 420 × 445 (mm); weight, 13.5 kg; main body sample holder, T25 flask insertion, maximum six flasks, Atmosphere inside the chamber: 37 °C, 5% CO2.

Gravite (Space Bio-Laboratories Co., Ltd., Hiroshima, Japan) is a simulated microgravity generator (Fig. 6) that uses simultaneous rotation of the chamber on two axes at a constant angular velocity to uniformly distribute, and cancels the cumulative gravity vector to simulate microgravity^34^. Initially, 1 × 10^6^ SNU-790 and SNU-80 cells were sub-cultured under 1 G conditions for 24 h. After the subculture period, the same number of cells (1 × 10^6^ cells) for each cell line was re-seeded into T-25 cell culture flasks (Corning) with RPMI-1640 media (Cytiva), supplemented with 10% FBS (Cytiva), and 1% penicillin-streptomycin (Thermo Fisher Scientific) in a humidified atmosphere of 5% CO_2_ at 37 °C. Cells were cultured until day 7 without changing the medium. Cell morphology and cell number via counting were confirmed after 24, 48, 72, and 120 h following re-seeding and culturing cells. Cell morphology imaging and cell counting were confirmed based on the adherent cells at the bottom of the T25 flask. Phase-contrast microscopy using Nikon eclipse TS100 (Marshall Scientific, Hampton, NH, USA) with 4×, 10×, 20×, and 40× objective lenses was used for observation of the cell morphology (Fig. 1 and Fig. 2). The cultured cells were enumerated using Life technology countess II (Thermo Fisher Scientific).

The culture conditions of the experimental and control groups in the Gravite are depicted in Fig. 6. The SNU-790 and SNU-80 cells, which had been sub-cultured for 24 h, were divided into T25 flasks at a density of 1 × 10^6^ cells. In the experimental group, the T25 flask was positioned in the Gravite sample holder, whereas in the control group, the T25 flask was placed at the bottom of the Gravite chamber. Both the experimental and control groups were subsequently cultured for a duration of 120 h. Placing the T25 flask in the control group at the bottom of the Gravite chamber established equivalent conditions that could potentially impact cell culture, aside from the effects of gravity. This experimental setup was employed to ensure a consistent and controlled environment for both groups, facilitating accurate comparisons and observations.

### RNA extraction, quality control, and cDNA microarray

After culturing the cells for 5 d, the supernatant was removed from the T25 flask, and adherent cells were washed twice with phosphate buffered saline (Cytiva). Subsequently, 1 mL of 0.25% Trypsin-EDTA buffer was added, and incubated for 1 − 2 min; the growth medium was added, and the cultured cells were transferred to a 15 mL tube and centrifuged at 4 °C and 3000 rpm (1952 × *g*) for 3 min using Hanil combi R515 (Hanil Scientific Inc., Gimpo, Korea). After centrifugation, the supernatant was aspirated, leaving only the pellet.

The pellets were sent to Macrogen (Macrogen, Inc., Seoul, Korea) for RNA extraction, which was performed as follows. For homogenization prior to RNA extraction, the cultured cells were uniformly immersed in lysis buffer using TransZol (TransGen Biotech Co., Ltd., Beijing, China) and disrupted. The lysate was transferred to a microcentrifuge tube and incubated for 5 min at room temperature (18–23 °C) after the visible precipitate had dispersed. Chloroform (Merck KGaA, Darmstadt, Germany) was added to the microcentrifuge tube at a rate of 0.2 mL per 1 mL of TransZol. The tube was shaken vigorously for 15 s, incubated at room temperature (18–23 °C) for 3 min, and then centrifuged at 13,000 × *g* for 15 min at 4 °C. Next, the colorless upper aqueous phase containing RNA was transferred to a new RNase-free tube. Isopropanol (Merck KGaA) was added to the RNase-free tubes at a ratio of 1 mL of isopropanol per 1 mL of TransZol. The tube was incubated at room temperature (18–23°C) for 10 min and centrifuged at 13,000 × *g* for 10 min at 4 °C. Following centrifugation, the supernatant was discarded. After adding 1 mL of 80% ethanol (Thermo Fisher Scientific), the tube was mixed vigorously and centrifuged at 13,000 × *g* for 5 min at 4 °C. Following centrifugation, the supernatant was discarded and the step was repeated by adding 1 mL of 80% ethanol to the tube. The tube was mixed by inverting and centrifuged at 13,000× *g* for 10 min at 4 °C. Again, following centrifugation, the supernatant was discarded. After air-drying the RNA pellet for approximately 5 min, it was dissolved in distilled water (Thermo Fisher Scientific) and incubated at 55 − 60 °C for 10 min.

Comprehensive RNA quality control measures were conducted, including purity and quantity assessment using a NanoDrop (Thermo Fisher Scientific). Absorbance measurements at 260 nm were employed to quantify RNA. Purity values of 1.7–2.0 were considered indicative of relatively pure RNA. An RNA integrity evaluation was performed using an Agilent Technologies 2100 Bioanalyzer (Agilent Technologies, Santa Clara, CA, USA) with an RNA Integrity Number value ≥8. Additionally, RNA quantity was assessed using a Quantus Fluorometer with Quant-iT microRNA Assay Kit (Thermo Fisher Scientific). RNA integrity was evaluated using an Agilent Technologies 2100 Bioanalyzer with Small RNA Chip (Agilent Technologies).

GeneChip Human Gene 2.0 ST Array (Thermo Fisher Scientific) was used in this study. cDNA was synthesized using the GeneChip WT (Whole Transcript) Amplification kit (Thermo Fisher Scientific), as described by the manufacturer. The sense cDNA was then fragmented and biotin-labeled with terminal deoxynucleotidyl transferase (TdT) using the GeneChip WT Terminal labeling kit (Thermo Fisher Scientific). Approximately 5.5 μg of labeled DNA target was hybridized to the Affymetrix GeneChip Array (Affymetrix Inc., Santa Clara, CA, USA) at 45 °C for 16 h. Hybridized arrays were washed and stained on a GeneChip Fluidics Station 450 (Thermo Fisher Scientific) and scanned on a GCS3000 Scanner (Affymetrix Inc.). The probe cell intensity data computation and a CEL file generation were performed using Affymetrix GeneChip Command Console Software (Affymetrix Inc.).

### Statistical analysis

In the DEG analysis, raw CEL files were processed using the Affymetrix Power Tools program (Affymetrix Inc.) with the Robust Multichip Analysis (RMA) algorithm to extract normalized signal intensity^35^. Filtering was then applied to retain probe sets that were not related to genes or transcripts, but rather contained main or consensus probes only. Subsequently, the fold change of the filtered probes was calculated, and the Local-pooled-error (LPE) test was performed to obtain LPE *P*-values^36^. However, the LPE *P*-value was not considered for selecting significant probes for Functional Analysis. Instead, only the fold change was considered (absolute value of fold change ≥ 1.5) to ensure a sufficient number of significant probes for further analysis. For a DEG set, hierarchical cluster analysis was performed using complete linkage and Euclidean distance as a measure of similarity. Gene-Enrichment and Functional Annotation analysis for a significant probe list was performed using Gene Ontology (http://geneontology.org) and KEGG (http://kegg.jp). Enrichment results were analyzed for GO terms with an adjusted *P*-value < 0.05 for each GO category. Considering that the statistical significance of GO terms with very small or large term sizes may be exaggerated, size filtering was performed on the top 20 GO terms that satisfy a term size of ≥ 10 and ≤ 500. All data analysis and visualization of DEGs were conducted using R 3.3.2 (www.r-project.org; The R Foundation, Indianapolis, IN, United States).

## Data Availability

As a principle of the hospital, the original data used in this study cannot be disclosed. All relevant data can be provided through communication with the corresponding author.
